# Editorial: Towards dependable brain computer/machine interfaces for movement control

**DOI:** 10.3389/fnhum.2023.1186423

**Published:** 2023-04-04

**Authors:** Gernot R. Müller-Putz, Jennifer L. Collinger, Reinmar J. Kobler

**Affiliations:** ^1^Graz BCI Lab, Institute of Neural Engineering, Graz University of Technology, Graz, Austria; ^2^BioTechMed, Graz, Austria; ^3^Rehab Neural Engineering Labs, University of Pittsburgh, Pittsburgh, PA, United States; ^4^Departments of Physical Medicine and Rehabilitation and Bioengineering, University of Pittsburgh, Pittsburgh, PA, United States; ^5^RIKEN Center for Advanced Intelligence Project (AIP), Tokyo, Japan

**Keywords:** movement control, brain-computer interface, brain-machine interface, decoding, feedback, closed-loop control

## Introduction

Movement control is among the top brain-computer interface (BCI) application scenarios for potential end-users. Movement control could involve moving a computer cursor on a screen, controlling a robotic arm, or reanimating one's own limbs. Current approaches vary in numerous factors, including signal modality [ranging from single unit spiking activity up to scalp-based electroencephalographic (EEG) recordings], control strategies, decoding algorithms, artifact mitigation, and feedback modalities (e.g., visual or somatosensory). In spite of this diversity, there are shared key challenges that must be solved in order to deliver dependable assistive technology that will meet the needs of end-users. In addition to safety, availability, and portability, a dependable BCI has to eliminate or alleviate time-consuming system (re-)calibration processes, be independent of artifacts introduced by body movements or external stimulations, and generalize to various movement tasks with different dynamics.

The goal of this Research Topic was to share recent advances among diverse approaches and, thereby, facilitate exchange between groups and research directions. We aimed for a scope ranging from original neuroscientific studies investigating neural correlates of goal-directed movements to neural engineering applications toward dependable BCIs for movement control.

## Research Topic coverage

In this Research Topic we collected four original scientific papers which shed light to specific aspects of the quest to develop dependable BCI technology. All of the articles use non-implanted neural recording technologies. Three manuscripts use EEG recordings of brain activity, while the fourth manuscript uses surface electromyography (sEMG) recordings of muscle activity during a large variety on hand movements.

BCIs rely on an understanding of the neural correlates of movement at the level of the brain, however in order to restore natural movement, it is also important to understand the peripheral activity of these movements, i.e., the EMG activity, to be able to further connect movement generation with movement execution in all levels.

To enhance accuracy and stability of gesture recognition based on sEMG, Peng et al. proposed a method combining feature selection and Ensemble Extreme Learning Machine (EELM) to improve the recognition performance based on sEMG signals. The authors compared the suggested methods with classical ensemble classifiers on a data set with 52 different hand movements from 10 able-bodied participants. Ultimately, sEMG could be used to classify these different movements with >75% accuracy.

Many BCIs rely on motor imagery to generate consistent patterns of neural activity that can be used as a control signal, however imagery performance can vary widely from person-to-person. In earlier studies, it was found that resting-state alpha-band power, also described as mu-band power, is positively correlated with motor imagery-based BCI performance. Zhou et al. conducted a study to evaluate whether neurofeedback training based on up-regulation of the alpha band relative power had an effect on motor imagery BCI performance. A general finding was that such alpha neurofeedback training could successfully help study participants increase their alpha rhythm power and improve their motor imagery BCI performance. Also, on an individual level, participants with increased alpha power had a larger performance improvement.

The next two papers in the collection, Plucknett et al. and Srisrisawang and Müller-Putz studied feature representations to aid subsequent decoding pipelines with regard to performance increases and interpretation.

Plucknett et al. investigated how various combinations of feature extraction and metric learning algorithms affected feature representations in a self-paced, binary center-out movement task. As a supervised dimensionality reduction technique, the goal in metric learning is to project data in a low-dimensional space that maintains discriminative information. In their study with a publicly available EEG dataset comprising two subjects, they report that the considered metric learning algorithms did not improve classification performance but were useful to identify the importance of EEG channels encoding discriminative information. Their findings confirmed the importance of low-frequency (<5Hz) EEG activity in movement discrimination tasks.

The majority of EEG BCI studies for hand trajectory decoding rely on low frequency activity using features in sensor space, rather than source space which estimates the activity at locations in the brain from the sensor activity using inverse modeling techniques. In the study of Srisrisawang and Müller-Putz, they tackle the problem of co-linearity due to the higher number of dimensions in the source space by two folds: (i) they selected signals in predefined regions of interest (ROIs) and (ii) they applied dimensionality reduction techniques like computing the mean (Mean), principal component analysis (PCA), and locality preserving projections (LPP) in pre-defined ROIs. Also the effect of using individual head models compared to a template model was investigated. Statistical tests revealed no significant differences between the source-space and sensor-space approaches when decoding reach velocities. Similarly, there was no significant difference between subject-specific and template head models. While no significant differences in performance were noted for correlation-based decoding of velocity, further investigations are still necessary when it comes to other decoding approaches such as a classification task.

All articles in this collection addressed important issues concerning BCIs for movement control. Three papers investigated feature extraction and classification methods from EMG (Peng et al.) and EEG (Plucknett et al.; Srisrisawang and Müller-Putz) and one EEG-based neurofeedback training (Zhou et al.). Despite the progress made in these and other works, further research is required toward establishing dependable BCIs for movement control.

## Author contributions

All authors listed have made a substantial, direct, and intellectual contribution to the work and approved it for publication.

